# Spontaneous template beats: A physiological clue for effective left bundle area pacing

**DOI:** 10.1016/j.ipej.2026.03.013

**Published:** 2026-03-18

**Authors:** Sampath Kumar Madapati, Krishna Prasad Akkineni, Mohan Prasad Akkineni, Shunmuga Sundaram Ponnusamy

**Affiliations:** a Department of Cardiology, Apollo Hospitals, Hyderabad, India; b Department of Cardiology, PGIMER, Chandigarh, India; c Department of Cardiology, Velammal Medical College Hospital, Madurai, India

**Keywords:** Left bundle area pacing, Template beat, Left bundle capture beat, Proximal conduction system disease, Biventricular pacing

## Abstract

Recent evidence suggests that left bundle area pacing (LBAP) is non-inferior to conventional cardiac resynchronization therapy (CRT) using biventricular pacing (BVP). In addition, LBAP directly recruits the native conduction system, thereby addressing several procedural limitations inherent to BVP. Despite these advantages, electrocardiographic (ECG) analysis rarely provides specific clues to identify patients who may benefit most from LBAP. We report a case in which a spontaneous “template beat” or “left bundle capture beat” was observed on the baseline ECG and propose this finding as a potential novel marker for identifying patients likely to respond favourably to this therapy.

## 1 Introduction

LBAP is emerging as an effective alternative to BVP for CRT. However, ECG markers to identify patients who may benefit most from LBAP remain limited. In this report, we describe a novel marker: the presence of a spontaneous left bundle capture beat on baseline ECG, which may help predict favourable response to LBAP.

## 2 Case presentation

**Fig. 1:**
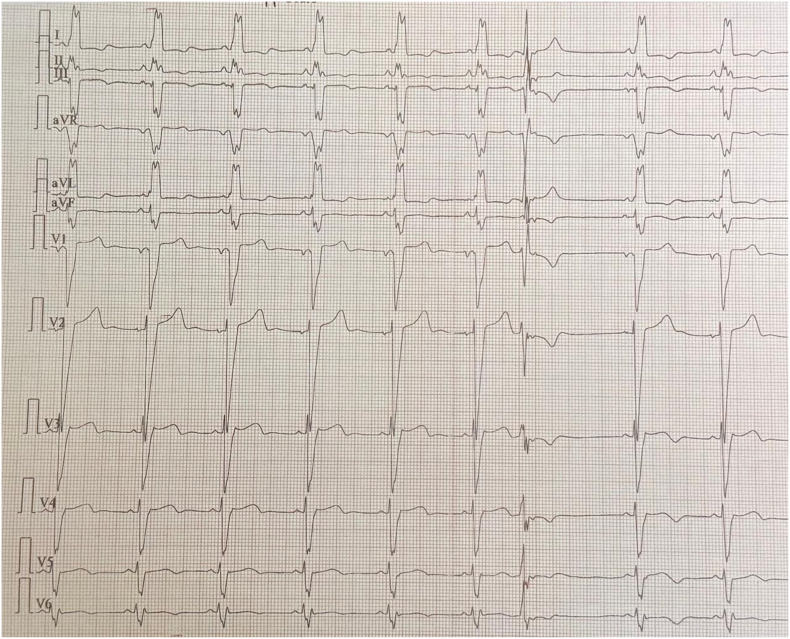
12 Lead ECG taken during follow up visit.

A 47-year-old female, with no known comorbidities, came to the hospital with worsening shortness of breath since the last 6 months. Echocardiography was suggestive of global hypokinesia with an ejection fraction of 25 percent. Coronary evaluation did not reveal any significant stenoses. Despite optimal guideline directed medical therapy (GDMT), she remained in NYHA III. Her ECG during follow up visit is shown in Fig. 1 which demonstrates sinus rhythm with left bundle branch block (LBBB) with dominant S waves in V1 and absence of q waves in the lateral leads.

The QRS duration is significantly prolonged with a duration of around 180msec, and meets Strauss criteria for true LBBB with duration >130msec (in women), QS complex in lead V1 and mid QRS notching in I and aVL [1]. Another important finding in this ECG is a QRS complex with right bundle branch block (rSR’ in lead V1, slurred S in lateral leads) morphology and a duration of 120 ms, recorded as the 7th beat. Since this beat is not preceded by a P wave and shows RBBB morphology, it is unlikely to represent an atrial premature contraction (APC) or a junctional beat with aberrancy. Therefore, this RBBB morphology QRS is a ventricular premature contraction (VPC) originating from the left ventricle. In our case, the presence of a VPC with RBB delay pattern (rSR’ in lead V1) and tall R-waves in leads II and aVF, spontaneously occurring on the baseline ECG with underlying LBBB suggests that this VPC is originating from the proximal left bundle. This finding indicates that the LBBB in this patient is most likely due to a focal block in the proximal conduction system, rather than diffuse distal disease or intrinsic myocardial pathology thus having important therapeutic implications.

Extensive evaluation did not reveal any aetiology and cardiac MRI was negative for significant LGE/fibrosis. Despite guideline directed medical therapy, the patient remained in NYHA III. Since she had LBBB morphology with QRS duration of 180msec, she was planned for biventricular pacing. However, the VPC morphology with narrow QRS and ‘M pattern’ (rSR’ in lead V1, Fig. 1) was suggestive of proximal conduction system disease induced cardiomyopathy. Hence left bundle branch pacing was considered over biventricular pacing to provide better synchronization therapy [2].

**Fig. 2:**
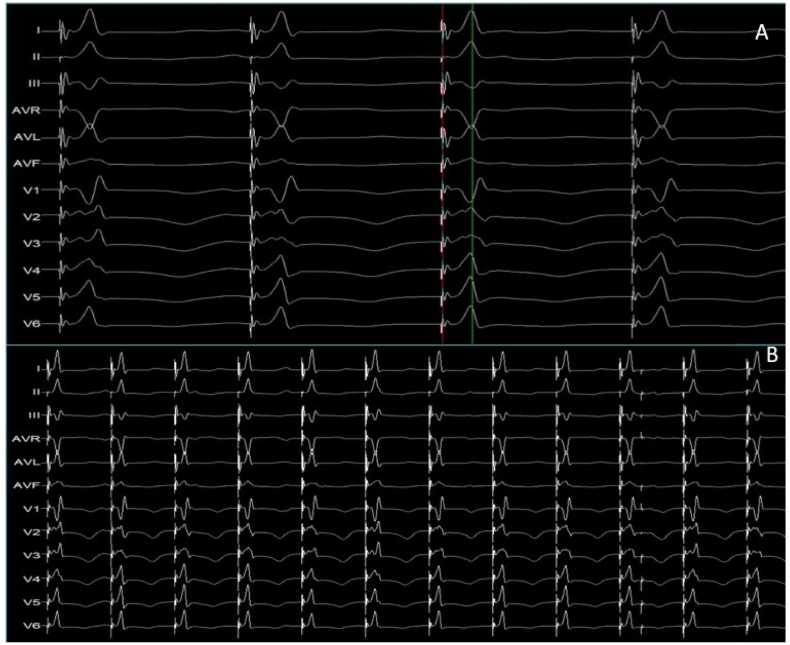
Panel A showing initial screwing of the lead causing QR pattern in V1 with LVAT of 70 seconds. Panel B demonstrating further rotations of the lead which led to narrower QRS and the ‘M beat’ morphology in V1 suggestive of left bundle capture.

While deploying the pacing lead (Selectsecure lead) into the septum, we first encountered a RBB delay pattern with QR in V1 (Fig. 2A). The LVAT was about 70 seconds.

**Fig. 3:**
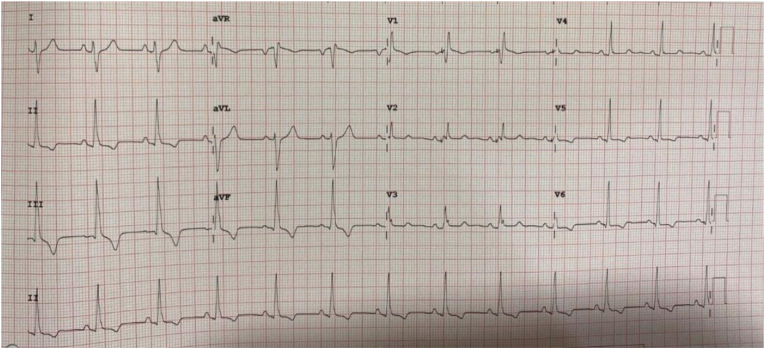
Final QRS morphology after optimizing AV delay. The QRS is narrow and the morphology resembles the ‘M beat’.

Further lead rotations resulted in a narrower QRS with an rSR’ pattern, resembling the “template beat” or a left bundle capture beat (Fig. 2B). Pacing thresholds were satisfactory. The paced beats closely matched the spontaneous VPCs recorded on the ECG, confirming that the VPCs originated from the proximal left bundle branch fibers. Optimization of the AV delay led to additional narrowing of the QRS duration while maintaining the characteristic “M beat” morphology (Fig. 3). The ejection fraction improved to 45% on follow up with marked improvement in symptoms. She has been under regular follow up for the past year without any relapse or worsening of symptoms.

## 3 Discussion

Current guidelines recommend cardiac resynchronization therapy (CRT) with biventricular pacing (BVP) as a Class I indication for patients with reduced ejection fraction (EF < 35%), persistent symptoms despite GDMT, and LBBB with a QRS duration >150 ms [3]. However, BVP-CRT is limited by a non-responder rate of approximately 30%, the intrinsic limitations of ventricular cell-to-cell conduction, and procedural challenges such as the inability to access an appropriate target vein, phrenic nerve stimulation, or the presence of intact atrioventricular conduction. Conduction system pacing offers a physiological alternative to conventional BVP-CRT by directly engaging the native conduction system. This approach not only restores or maintains bundle branch conduction but also preserves a narrow QRS complex.

Ponnusamy et al. astutely pointed a novel method of LBB area pacing guided by VPC's. They demonstrated that as the septum was pierced by the pacing lead, VPCs were induced which changed in morphology from QS pattern to qR or rSR’ pattern, with a duration less than 130msec, denoting LBB capture and proximity to the left bundle [4]. These VPC's were termed as left bundle capture beats or ‘template beats.’ At this point, the lead was secured in the septum, as further turns increased the risk of perforation. The paced morphology simulated the ‘template beat’ thus proving that this unique VPC was actually a marker of LBB capture [4].

‘Template beats’ are generated from Purkinje fibres due to mechanical trauma induced by the pacing lead as it approaches the LBB area. Ponnusamy et al. demonstrated that the ‘template beats’ guided LBB area pacing was associated with lesser fluoroscopy time, narrower paced QRS, shorter peak left ventricular activation time, minimal myocardial injury and reduced risk of septal perforation [5]. These beats were observed in about 90% of the patients undergoing LBB area pacing and had sensitivity of 96% and specificity of 97% to denote capture of LBB area [6]. Combining the use of ‘template beats’ with other indicators of LBB capture like LBB potential, retrograde His potential and abrupt shortening of stimulus artifact to LVAT >10 ms further optimized the results in these patients.

In our case, such ‘template beats’ occurred spontaneously on baseline ECG likely due to enhanced automaticity of Purkinje fibres. These spontaneous VPC's on baseline ECG have rarely been documented and indicate that the left bundle below the block is still electrically excitable and functional, even though the antegrade His-Purkinje conduction is blocked. This finding strongly predicts a favourable response to LBB area pacing, since capture of the left bundle distal to the block is feasible.

## 4 Conclusion

To conclude, RBBB morphology VPCs (template beats) in a patient with baseline LBBB may serve as a novel marker of focal proximal left bundle block and can predict a favourable response to LBB area pacing. Recognizing these ‘template VPC's’ on routine ECG can guide therapy and improve outcomes.

## Patient consent

Informed consent was obtained from the patient for publication of case details.

## Ethical clearance

Clearance was received from the Ethics committee for publication and submission of this case report.

## Funding sources

This research did not receive any specific grant from funding agencies in the public, commercial, or not-for profit sectors.

## Declaration of competing interest

The authors declare that they have no known competing financial interests or personal relationships that could have appeared to influence the work reported in this paper.

## Data Availability

All data pertaining to the case is available with the corresponding author and will be shared upon reasonable request.
